# Assessing fidelity measurements in school-based anxiety, depression and suicide prevention programs: a systematic review

**DOI:** 10.1186/s12889-025-24219-5

**Published:** 2025-09-01

**Authors:** Dominique van Pelt, Saskia Mérelle, Kristel Jenniskens, Daan Creemers, Jan Spijker, Leonie van Vuuren, Femke van Nassau, Arne Popma, Sanne Rasing

**Affiliations:** 1 Behavioural Science Institute, Radboud University, Nijmegen, Netherlands; 2 113 Suicide Prevention, Amsterdam, Netherlands; 3 GGZ Oost Brabant, Boekel, Netherlands; 4 Pro Persona, Nijmegen, Netherlands; 5 Department of Public and Occupational Health, Amsterdam UMC Location Vrije Universiteit Amsterdam, Amsterdam, Netherlands; 6 Health Behaviours & Chronic Diseases, Amsterdam Public Health Research Institute, Amsterdam, Netherlands; 7 Child and Adolescent Psychiatry & Psychosocial Care, Amsterdam, Netherlands

**Keywords:** Implementation fidelity, School-based anxiety depression and suicide prevention programmes, Adherence, Dose, Responsiveness, Quality of delivery, Differentiation

## Abstract

**Background:**

To ensure the effectiveness of school-based anxiety, depression and suicide prevention programmes, implementation fidelity, defined as the degree to which interventions are implemented as intended, is crucial. A comprehensive overview of fidelity measurements in these programmes is currently lacking, limiting the ability to compare and improve implementation efforts. This is particularly challenging in the context of scaling up prevention programmes, where ensuring both high-quality implementation and widespread adoption often proves insufficiently successful. With this review we aimed to (1) examine the extent to which fidelity measures were used and reported in existing studies of school-based anxiety, depression and suicide prevention programmes; (2) identify which fidelity components were measured; and (3) evaluate the quality of the fidelity measurements.

**Methods:**

A systematic search was conducted across PubMed, PsycINFO, Medline, and ERIC using an AND-combination of search terms related to schools, adolescents, depression and interventions. Two researchers screened the selected articles, with discrepancies resolved by a third. Pre-defined inclusion criteria were used based on school-based prevention programmes, controlled trials, and psychological intervention techniques. For data extraction, fidelity components were extracted together with the methods for fidelity measurement. The quality of the measurements was assessed using criteria used in earlier fidelity research.

**Results:**

Of 13,131 identified articles, 190 met our inclusion criteria. Of these, 72 (38%) measured at least one fidelity component, most commonly adherence (57, 79%), followed by responsiveness (24, 33%), dosage (19, 26%), and quality of delivery (9, 13%). Programme differentiation was not measured. The quality of fidelity measurements was most frequently moderate for adherence (65%), dosage (53%), and quality of delivery (56%), but low for responsiveness (79%).

**Conclusion:**

Most school-based anxiety, depression, and suicide prevention programmes lack fidelity measurements, and existing assessments are often of moderate to low quality. To improve fidelity measurement, future research should prioritise the development of standardised methods with clear definitions and practical tools for assessing fidelity components. Embedding fidelity as a central element in study designs is essential to better link implementation efforts to intervention outcomes and to fully understand and optimise programme effectiveness, while ensuring that fidelity can be feasibly monitored in real-world settings.

**Supplementary Information:**

The online version contains supplementary material available at 10.1186/s12889-025-24219-5.

## Background

In recent years, rates of depression and suicide have risen to levels that demand urgent attention. Research has shown an increase in the number of adolescents with depression globally [1], which is concerning as depression or depressive symptoms during adolescence can lead to a higher chance of having psychiatric, other health, and social challenges later in life [2]. Furthermore, the mental health of young adults has deteriorated in the past two decades, reported as a rising prevalence of adolescents with anxiety, depression, and suicide problems [3]. Consequently, these challenges are associated with an increased risk of self-harm and suicidal behaviour [4]. Therefore, the implementation of effective prevention strategies is crucial.

To address the issue of depressive symptoms in youth, several school-based depression prevention programmes have been developed. These are typically primary and secondary prevention programmes, aiming at prevention of the onset of mental health issues or interfere early in at-risk adolescents. Schools have become a key setting for mental health education as schools are compulsory for adolescents and have the potential to reach large numbers of students. Whether within or outside the school setting, prevention programmes have been shown to effectively reduce the risk of developing a depressive disorder. This is supported by a systematic review and meta-analysis conducted by Werner-Seidler et al. [5], which found that school-based programmes are effective in reducing the risk of depression and anxiety, among adolescents. Research by Gijzen et al. [6] also found that school-based prevention programmes have a small effect on reducing the risk of suicidal thoughts and behaviours (STBs). Besides depression and STBs, anxiety is important to consider, given its link to suicidal thoughts and behaviours [7]. By addressing both anxiety and depression, school-based prevention efforts may enhance their relevance and impact, ensuring broader coverage of mental health challenges faced by youth. Therefore, the focus of this review is on school-based primary and secondary prevention programmes targeting anxiety, depression, and suicide prevention, as these are among the most prevalent issues in adolescence and are often addressed using similar psychological approaches. Besides that, we ensure greater conceptual and methodological homogeneity by limiting the scope to internalising problems. This allows for a more targeted analysis of fidelity measurement practices.

Much research has focused on the effectiveness of prevention programmes, but few studies have evaluated how such programmes are implemented [8]. Prior research has indicated that evaluating the implementation of a prevention programme can enhance its efficacy [9]. A systematic review of 31 school-based mental health programmes found that implementation fidelity components were positively associated with the outcomes of the intervention in 40% of the 273 associations examined. This shows that fidelity is an important determinant of programme effectiveness [10]. Furthermore, in light of the global decline in adolescent mental health, there is an urgent need for the implementation of such programmes at the national level. However, when evidence-based programmes are introduced into real-life settings, the manner in which the programme was taught in the study does not always align with the actual implementation. Scaling up these programmes presents a significant challenge, as it requires balancing fidelity to the evidence-based design with the practical and contextual realities of diverse environments [11]. In practice, ensuring high-quality implementation at scale often proves insufficiently successful, limiting the potential impact of these interventions.

Fidelity refers to the extent to which a programme is being delivered as originally designed and intended by the programme developers [12]. It is instrumental in understanding the extent to which the outcomes can be attributed to the intervention. When an intervention is delivered with low fidelity, it means that there is high heterogeneity in the delivery. This is an unwanted source of variability in the testing of the effectiveness of intervention. With fidelity measurements, insight is provided into the methods used to implement a programme but also into what alterations were made to ensure better adaptation to a particular setting. While strict adherence to the original design is considered crucial, there is an ongoing debate in the literature that some level of deviation may be necessary to adapt to different settings [13]. Besides that, the measurement of fidelity is a challenging task, due to the many frameworks that have been proposed for this purpose [9, 12, 14]. A review studying the fidelity measurements in smoking cessation programmes found that there is also considerable variability in which components of fidelity are measured and in the methodology employed to measure them, across studies. While some studies make use of the responsiveness of participants to a programme, others put more emphasis on the dose delivered to a group of participants [15].

A systematic review by Feiss et al. [16] investigated the effectiveness of school-based programmes in mental health problems and also looked into programme dose as a moderator. However, this review did not systematically assess all components of fidelity and their quality. This review aims to address this gap by giving a comprehensive overview of the use of fidelity measurements and their quality in school-based anxiety, depression and suicide prevention programmes. Other fields have also examined fidelity assessments in prevention programmes. For example, Begum et al. [15] explored fidelity measurements in smoking cessation programmes. Furthermore, in the field of obesity prevention, Schaap et al. [17] conducted a review of fidelity measurements in obesity prevention programmes with the objective of gaining an overview of how fidelity is assessed in that specific field. We partly replicated their approach for the field of school-based anxiety, depression and suicide prevention programmes. Our systematic review aimed to (1) examine the extent to which fidelity measurements were used and reported in existing studies of school-based anxiety, depression and suicide prevention programmes; (2) identify which fidelity components are measured; and (3) evaluate the quality of the fidelity measurements. This information is crucial for facilitating the implementation of school-based anxiety, depression and suicide prevention programmes.

## Methods

A comprehensive search strategy was conducted according to the Joanna Briggs Institute (JBI) methodology for systematic reviews [18] and reported following the based on the Preferred Reporting Items for Systematic Reviews and Meta-Analysis (PRISMA) 2020 statement [19] to ensure the literature review was thorough and unbiased (Additional file 1, Table S1). This systematic review was preregistered in PROSPERO (ID no. CRD42024503835).

### Literature search

One author (DP) conducted the literature search in the following databases: PubMed, PsycINFO, Medline, and ERIC. The search strategy employed an AND-combination of search terms related to the setting (e.g. ‘school’), group (e.g. ‘adolescen*’), symptoms (e.g. ‘depress*’, ‘anxious’, ‘suicidality’), and programme (e.g. ‘interven*’). The complete search strategy used for each database, along with the corresponding number of articles retrieved, is provided in Additional file 2. It should be noted that no limitations were placed on the timeframe during this phase. All included articles were transferred to EndNote and checked for duplicates.

### Screening process

The following inclusion criteria were applied: (1) school-aged children and adolescents with an age range of 10–23 years old, (2) the programme focused on depression, anxiety or suicide-related problems of young people, (3) the preventive intervention was offered in a school setting, (4) the prevention programme was based on psychological techniques with a theoretical or empirical basis and includes evidence-based components or standardized recommendations to reduce the risk of or manage mental health problems, (5) the study design was (quasi-)experimental, (6) only peer-reviewed articles in the English language were included. Studies were excluded when the programme described a clinical treatment programme or a non-verbal method/protocol primarily based around activities (e.g., colouring), and when the publication was a dissertation, conference abstract, study protocol, or other form of grey literature. The focus on (quasi-)experimental designs is chosen as fidelity should ideally be addresses during controlled evaluation of a programme to allow a valid interpretation of intervention outcomes. Two researchers (DP and KJ) were involved in the screening processes. The records were initially screened independently by the reviewers based on title and abstract and subsequently by full text. In the event of a discrepancy, a third reviewer was consulted (SM). The screening process was conducted using Rayyan, a collaborative platform that facilitates the systematic review process by allowing researchers to import, screen, tag, and analyse large sets of references and allowed to track agreements and conflicts between reviewers.

### Presence fidelity measurement

Each included study was checked by DP to assess if one or more fidelity components were measured (i.e. Adherence; dosage; quality of delivery; responsiveness; and programme differentiation). In this review, the fidelity components were based on the research of Dusenbury et al., [9], who expanded upon a previous framework by Dane and Schneider [20], and further informed by Carroll et al. [12]. These components are further defined in Table 1. This information was then noted in a designated excel document used for data extraction.

**Table 1:** Definitions of the fidelity components

Fidelity component*	Definition
Adherence	The extent to which a programme or intervention is delivered as it was designed, including prescribed content, methods and sequence of activities;
Dosage	Whether the frequency and duration of the intervention are as prescribed by its designers; typically expressed in terms of frequency, duration, or number of sessions. Dosage was coded as the frequency or duration of intervention exposure. Attendance records were not included, following the rationale that mere attendance does not guarantee content exposure or skill acquisition;
Responsiveness	Responsiveness refers to the degree to which both recipients of the intervention (e.g., students) and implementers (e.g., teachers or facilitators) are engaged with, committed to, and perceive value in the intervention;
Quality of delivery	Whether the manner in which a teacher, staff member or another trained professional delivers a programme contributes to the programme’s effectiveness, including factors as clarity, enthusiasm, and the ability to engage with the participants;
Programme differentiation	The extent to which a specific programme can be distinguished from other similar programmes based on its unique features.

*Definitions adapted from Dusenbury et al. [9], *based on Dane and Schneider* [20] *and further informed by Carroll et al.* [12]

### Fidelity components

For studies that included measurement of one or more of the aforementioned fidelity components, further data extraction was conducted. The data extraction of the articles was performed by DP and was based on the method proposed by Schaap et al. [17]. This entailed the comprehensive extraction of information from each study using a data extraction form in Excel. KJ independently extracted the fidelity components from a randomly selected subset (25%) of the articles that measured fidelity. The results obtained by the two researchers were compared, and any discrepancies were discussed until consensus on the final findings was reached. The remaining 75% of studies were evaluated again after this discussion to ensure consistency in the extraction of the fidelity components. The data extracted from the studies included the author, year of publication, country of study, age range of participants, name of the programme, aim, setting of the programme, programme provider, and the fidelity components; adherence, dosage, responsiveness, quality of delivery, and programme differentiation. Furthermore, additional measurement techniques for the fidelity components were identified. This included the specific methods used to measure fidelity such as logbooks, checklists and other observational tools. This part of the data extraction was conducted by DP. The extracted data was managed using Excel, and results were presented per study. If multiple articles evaluated the same programme, each article was evaluated separately for their presence and measurement of fidelity components. If the articles described different phases, e.g. short-term and long-term outcomes, these were analysed as separate studies and thus reported individually.

### Quality of fidelity measurement methods

In studies that reported on at least one fidelity component, an analysis was conducted on the procedure used to measure that component and the quality of the measurement, following the procedure described in Schaap et al. [17]. Since no official method existed for assessing the quality of fidelity components, they based their method on two reviews that also examined process evaluation data [8, 21]. This quality assessment was also conducted for this research by DP, as in the previous extraction step, KJ independently assessed a randomly selected subset (25%) of included articles that measured fidelity. The remaining 75% of studies were evaluated again after this discussion to ensure consistency in the application of the quality assessment criteria. The results were compared, and any discrepancies were discussed until a consensus was reached.

The assessment consisted of seven criteria that were used to evaluate each fidelity component (Table 2). For each fidelity component, the seven criteria were rated as either positive (+) or negative (-). It should be noted that the components ‘adherence’ and ‘quality of delivery’ varied regarding the second criterion, namely in their level of evaluation. This criterion examines whether a component was measured on more than one level; both the participant and the implementer. For responsiveness for instance, this could entail both a student feedback as well as a teacher self-report, which allows for a multi-level assessment. This reflects that fidelity can be the function of the interventionists’ behaviour as well as the recipients’ behaviour. Since adherence and quality of delivery can only be delivered by an instructor and thus evaluated at only one level, the second criterion regarding the level of evaluation was not applicable. Consequently, a score of ‘not applicable’ (NA) was applied for these two components on the second criterion. The quality assessment of each fidelity component was ultimately scored based on either six or seven criteria. A quality score was calculated for each component, ranging from 0% (where 6 or 7 criteria were scored negatively) to 100% (where 6 or 7 criteria were scored positively), with different calculations for the components of adherence and quality of delivery. Components were classified as high-quality (> 75% positive), moderate-quality (50–75% positive), or low-quality (< 50% positive) based on this score [17].

**Table 2:** Criteria for assessing methodological quality of fidelity components

		Fidelity component receives a positive score	Fidelity component receives a negative score
1	Model used for evaluation	If a theoretical framework or model for the evaluation was used and reported or referred to in the article	If no theoretical framework or model was used for the evaluation
2	Level of evaluation*	If the fidelity component was evaluated on two or more levels (i.e. teacher or student)*	If the fidelity component was evaluated on only one level (i.e. teacher or student)*
3	Operationalisation of fidelity component	If the fidelity component was defined or operationalised.	If only the name of the fidelity component was provided and not further defined or operationalised.
4	Data collection methods	If two or more techniques for data collection were used (triangulation).	If only one technique for data collection was used.
5	Quantitative fidelity measurements	If the measurement of the fidelity component was performed with adequately described methods	If the measurement of the fidelity component was not performed with adequately described methods
6	Frequency of data collection	If the fidelity component was measured on more than one occasion (e.g. pre, during, after delivery, such as post-intervention questionnaires and interviews)	If the fidelity component was measured on only one occasion.
7	Relation fidelity component and programme outcome assessed	If tested whether the fidelity component was related to programme outcomes.	If not tested whether the fidelity component was related to programme outcomes.

* Only applied to dosage, responsiveness and programme differentiation, as it is not possible to evaluate adherence and quality of delivery on two or more levels – i.e. only on instructor level

## Results

**Fig. 1:**
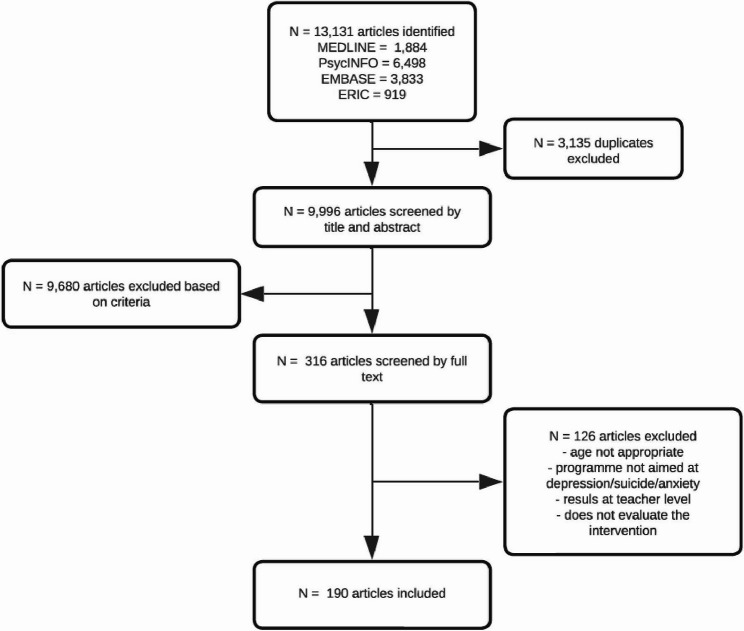
Flowchart of article selection process

After searching the relevant databases, a total of 13,131 articles were identified: 1,884 in Medline, 6,498 in PsycINFO, 3,833 in EMBASE, and 919 in ERIC. After removing duplicates using Endnote, 9,996 articles remained. Screening of titles and abstracts resulted in 316 included articles, and after full-text screening, the final selection consisted of 190 articles. The flowchart illustrating the article selection process is shown in Fig. 1.

### Presence fidelity measurement

**Fig. 2:**
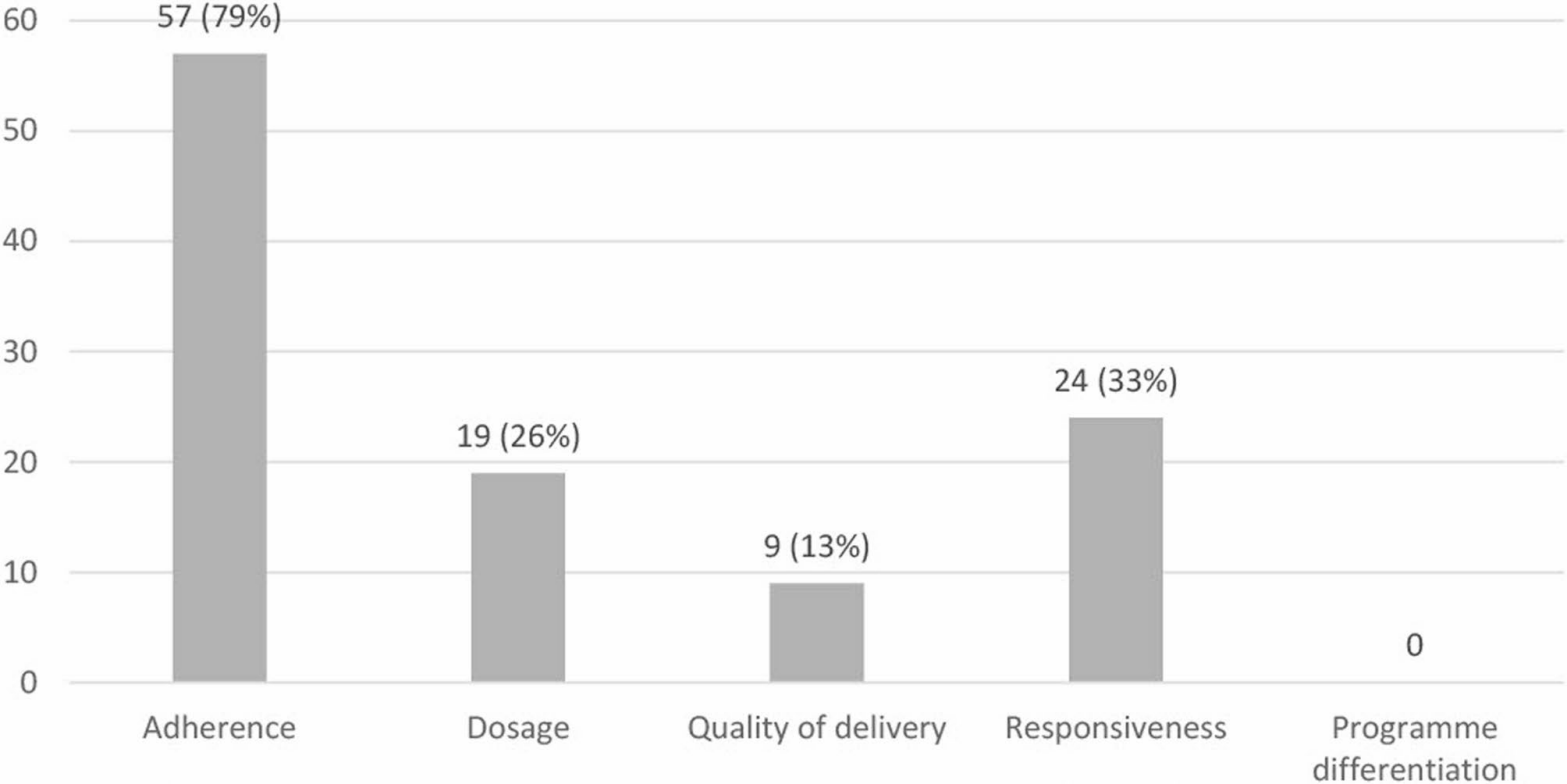
Measured fidelity components across included studies

**Fig. 3:**
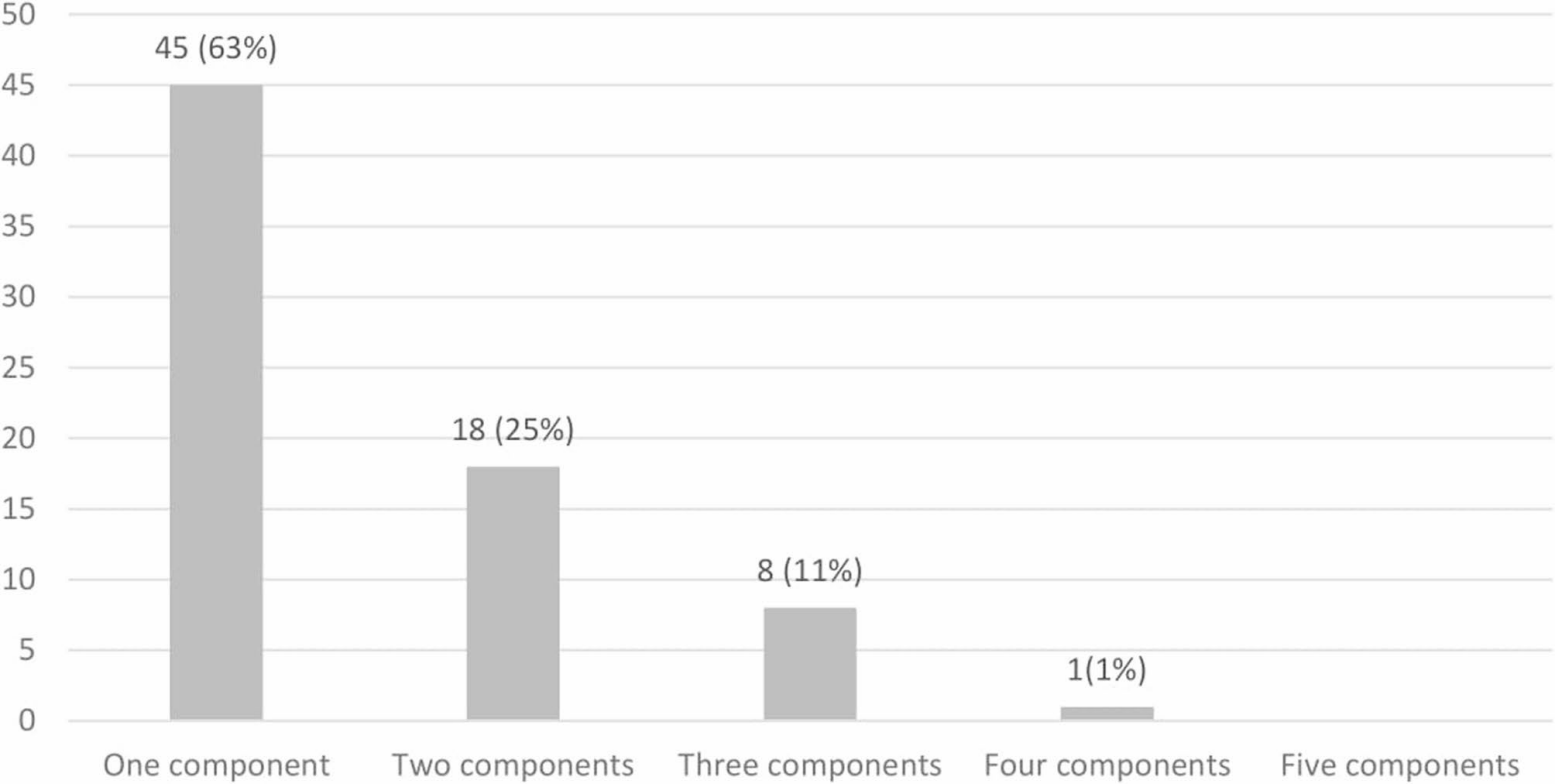
Number of fidelity components measured per study

A total of 190 articles met the inclusion criteria. One of our research aims was to examine the extent to which fidelity measurements were used and reported in existing studies of school-based anxiety, depression and suicide prevention programmes. Of the 190 articles included in the study, 72 were found to measure one or more components of fidelity (38%). The remainder of this study will focus on these 72 articles, as the primary objective is to analyse those that measure one or more components of fidelity. Across these studies, adherence was measured 57 times, dosage 19 times, quality of delivery 9 times, and responsiveness 24 times. Notably, programme differentiation was not measured in any of the studies (Fig. 2). The distribution of the measured fidelity components per study is shown in Fig. 3. For a detailed overview of the studies and their measured fidelity components, see Additional File 3: Table S3 and S4.

### Study characteristics

The studies were conducted in 22 different countries, with one study involving multiple countries. The United States accounted for the highest number of studies (*n* = 27), followed by Australia (*n* = 15). Among the articles that measured fidelity, the main focus was on depression-related problems (*n* = 32) or both anxiety and depression-related problems (*n* = 28). Most studies were conducted in secondary school (*n* = 66). Table 3 provides an overview of the study characteristics for the 72 included articles.

**Table 3:** Study characteristic

Study characteristics	Number of studies (*n*, %)	References
Country		
United States	27 (37.5%)	[22–48]
Australia	15 (20.8%)	[49–63]
Netherlands	3 (4.2%)	[64–66]
United Kingdom	3 (4.2%)	[67–69]
Spain	3 (4.2%)	[70–72]
Canada	2 (2.8%)	[73, 74]
Kenya	2 (2.8%)	[75, 76]
Norway	2 (2.8%)	[77, 78]
Chile	1 (1.4%)	[79]
Nigeria	1 (1.4%)	[80]
Lebanon	1 (1.4%)	[81]
Uganda	1 (1.4%)	[82]
New Zealand	1 (1.4%)	[83]
Japan	1 (1.4%)	[84]
Mauritius	1 (1.4%)	[85]
Ireland	1 (1.4%)	[86]
Israel	1 (1.4%)	[87]
Germany	1 (1.4%)	[88]
Malaysia	1 (1.4%)	[89]
Multiple countries	1 (1.4%)	[90]
Finland	1 (1.4%)	[91]
India	1 (1.4%)	[92]
Sweden	1 (1.4%)	[93]
Setting		
Primary school	4 (5.6%)	[54, 55, 63, 82]
Secondary school	66 (91.7%)	[22–51, 56–62, 64–81, 83–93]
Primary and secondary school	2 (2.8%)	[52, 53]
Main focus		
Suicide-related issues	3 (4.2%)	[26, 45, 65]
Depression-related issues	32 (44.4%)	[23, 27, 29, 33, 35, 37, 38, 40, 41, 50, 52, 56–60, 66, 68–70, 73, 74, 79, 80, 82, 83, 85, 88–90, 92, 93]
Anxiety-related issues	6 (8.3%)	[24, 25, 63, 77, 84, 86]
Anxiety and depression-related issues	28 (38.9%)	[22, 28, 30–32, 34, 42–44, 46, 48, 49, 51, 53–55, 61, 62, 64, 67, 71, 72, 75, 76, 78, 81, 87, 91]
Suicide, anxiety, and depression-related issues	3 (4.2%)	[36, 39, 47]

Some methods for the measurement of fidelity, such as observations and logbooks, were used in different formats across studies. For example; observations included live, video- and audio-recorded sessions, sometimes accompanied by a structured checklist or logbook, but not always. Similarly, interviews and questionnaires were used either to explore fidelity directly (e.g., by asking about adherence) or more generally to assess participant experience or engagement. In-depth qualitative analyses of such data fell outside the scope of this review. Therefore, the categorisation reflects the reported method rather than the depth or content of the analysis. Across the studies measuring fidelity, adherence was mostly assessed using observations and logbooks/checklists, while responsiveness was evaluated primarily through questionnaires. Dosage was measured using logbooks and observations, while quality of delivery was measured mostly through observations. Interviews were rarely used as a fidelity measurement method. For an overview of the characteristics of the methods used to measure fidelity, refer to Additional file 3: Table S5.

### Quality of fidelity measurement methods

There was considerable variability in the quality of fidelity measurements across the components. The component adherence was measured in 57 studies, of which the majority was rated as moderate quality (65%), 30% as low and 5% as high. For dosage, no studies were rated as high quality and the majority were qualified as moderate (53%). Quality of delivery wasn’t measured often and was mostly rated as moderate (56%), with no studies having high-quality rating. Responsiveness had the lowest overall quality, with most studies falling into the low category. These findings highlight that fidelity measurements, particularly for responsiveness and quality of delivery, were often of low quality, and no component was consistently measured with high quality. For an overview of the quality ratings of all fidelity components, see Table 4. To see how often each criterion was applied in the measurement of specific fidelity components, refer to Additional file 3: Table S6.

**Table 4:** Quality ratings of the measured fidelity component

Quality of the components	Number of studies (*n*,%)	References
Adherence		
Low (< 50%)	17 (30%)	[22–27, 64, 67, 70, 75, 76, 79–81, 83, 87, 90]
Moderate (50–75%)	37 (65%)	[28–41, 49–59, 65, 66, 68, 71–73, 77, 82, 86, 88, 89, 91]
High (> 75%)	3 (5%)	[42, 43, 78]
Dosage		
Low (< 50%)	9 (47%)	[22, 25, 31, 32, 44, 60, 74, 76, 85]
Moderate (50–75%)	10 (53%)	[29, 33, 34, 45–47, 61, 71, 72, 91]
High (> 75%)	0	
Quality of delivery		
Low (< 50%)	4 (44%)	[24, 27, 46, 75]
Moderate (50–75%)	5 (56%)	[47, 50, 51, 73, 76]
High (> 75%)	0	
Responsiveness		
Low (< 50%)	19 (79%)	[32, 42, 44, 45, 50, 51, 55, 62, 63, 71, 72, 74–76, 84, 85, 91–93]
Moderate (50–75%)	5 (21%)	[22, 48, 56, 57, 69]
High (> 75%)	0	

## Discussion

This study gives a comprehensive overview of the prevalence and quality of fidelity measurements in school-based anxiety, depression and suicide prevention programmes. Although earlier studies have emphasised the importance of fidelity in prevention programmes, ensuring that programme outlines are adhered to, the prevalence of fidelity measurements and their quality in this particular field has not been investigated to our knowledge. This review found that the majority of the studies into school-based anxiety, depression and suicide prevention programmes do not employ fidelity measurements and that, if employed, the quality of the measurements was often considered moderate or even low.

Similar to the findings of Schaap et al. [17], who examined fidelity reporting in school-based obesity prevention programmes, the majority of studies on anxiety, depression or suicide prevention programmes did not measure fidelity: of the 190 included articles, 38% of studies measured one or more components of fidelity. While this percentage indicates some recognition of the importance of fidelity in these studies, it also suggests that its measurement is still relatively uncommon, reflecting a partial but not yet widespread integration of fidelity measurement into this field of research.

The lack of consensus on the definition of fidelity and its components is a key challenge in fidelity research. For example, in the study by Cook et al. [67] the term “treatment integrity” is used instead of “adherence”, which demonstrates this variability in terminology and hinders the development of a standardised measurement. Different studies also conceptualise fidelity in different frameworks, such as Dusenbury et al. [9] who proposed a framework of five components of fidelity, while Carroll et al. [11] devised their own framework with the same components. With the presence of different frameworks of fidelity, it can be unclear for researchers to ascertain the precise procedures to measure an intervention’s fidelity.

To improve consistency in fidelity measurements, the field would benefit from establishing shared nomenclature and reporting guidelines and frameworks through consensus-building exercises. An example of such an approach is the COSMIN initiative, which uses specific procedures to develop standards for health measurement instruments. Methods used in this initiative are Delphi studies, consensus meetings with experts, and literature reviews. A similar procedure could support in defining fidelity components and guidelines which would ensure comparability between studies and also allowing for contextual adaptation for various settings in implementation research.

Besides conceptual inconsistency, there is great variation in the feasibility of measuring the components of fidelity. Adherence, for instance, includes more straightforward methods such as standardised protocols, checklists, or structured observations. In this review, adherence was the most frequently measured fidelity component, likely since it is the ‘bottom-line’ measurement of fidelity [12]. In contrast, the method to measure programme differentiation is less known and more difficult to execute. It was not measured in the included studies, possibly since there is lack of clarity on how to assess it. A method such as component analysis could help identify which elements are likely to have the most significant influence [12, 94].

In addition, the majority of studies focused on a single component and only one study measured four components [76]. While measuring more components can yield more insight into the functioning and effectiveness of the intervention it requires distinct measurement methods and data collection protocols which can be time-consuming. Besides that, for sustainable implementation, researchers, programme implementers and instructors should be responsible for fidelity measurements and need feasible methods to monitor fidelity in the real-world setting.

Our findings revealed that the quality of fidelity measurements was frequently rated as moderate or even low, and rarely as high. This can be attributed to the abovementioned factors. Moreover, in the absence of sufficient fidelity measurements, it is difficult to determine whether a lack of intervention effects is due to theory failure, meaning the programme itself is ineffective, or if they are influenced by the way the intervention was implemented, defined as programme failure. In a review by Begum et al. [15] that examined fidelity measurements in smoking cessation interventions, it was found that only 27% of the studies linked the fidelity data to the outcomes of the intervention. In the current review, the percentage of studies relating the outcome of the measurement of a fidelity component back to the outcomes of the intervention was 11%. This gap highlights a critical limitation, as it remains unclear whether the absence or weakness of observed outcomes of prevention interventions was a direct result of the intervention or influenced by the fidelity to the programme. It is however complex to relate fidelity components to clinical outcomes as it requires large sample sizes and advanced approaches. Particularly in the case of working with clustering effects such as classes and schools in nested designs [95].

Despite this limitation, interest in fidelity is growing. As the literature indicates, the predominant focus of researchers remains on outcome evaluation, with fidelity often considered retrospectively [96]. This lack of integration underscores the necessity of embedding fidelity measurements as a central component of intervention research to fully understand and optimise programme effectiveness.

An aspect related to fidelity and often discussed in the literature is adaptation - changes brought to an original design of an intervention by users or implementers, which is sometimes viewed as opposing fidelity [13]. However, adaptations can also be considered intentional strategies to enhance the fit of an intervention across diverse environments. Besides the assessment of fidelity components, the study by Pérez et al. [13] calls for a systematic documentation of adaptations and their potential impact on the outcomes of the intervention. They constructed a modified version of the framework by Carroll et al. [12] to incorporate adaptations made in order to capture how adaptation can affect fidelity and programme outcomes. Even though this fidelity-adaptation debate is particularly relevant in the context of school-based prevention programmes where adaptation may be needed, classical fidelity dimensions and conceptual frameworks do not address how to adapt an intervention while maintaining its effectiveness. A rigid position regarding fidelity can lead to innovations that are irrelevant or even inappropriate for certain users. In order to increase the change to fit a wider range of users, intervention need to be flexible and adaptable [12].

In addition to this, the effectiveness of a programme is not only dependent on its programme content and fidelity but also its ecological validity- the extent to which findings generalise to a real-life setting and an everyday environment. In order to ensure practical applicability, RCTs should also incorporate considerations of ecological validity. A study by Feiner et al. [97] investigated how outcomes of an intervention for autistic toddlers differ between different settings: one with family-selected materials and one with standardised toys. Results showed that the children tended to communicate more in a setting that was familiar to them. These findings show that in order to better reflect an interventions impact it is important to measure the outcomes of an intervention in real-life environments. This is essential for achieving a comprehensive understanding of and optimising the effectiveness of, the programme.

Finally, recent innovative developments have been made to improve both the quality and feasibility of fidelity monitoring. A study by Creed et al. [98] utilised artificial intelligence to generate automated feedback on therapist adherence and competence in cognitive behavioural therapy. This approach to fidelity monitoring has been shown to be effective in overcoming the limitations imposed by current methods, thereby facilitating scalable and accessible assessment of fidelity. The findings of this study show the potential of technology to integrate fidelity assessments into standard practices. Also school-based settings could benefit from innovations such as these. For instance by an automatic analysis of adherence to a session or student-teacher interactions through audio recordings.

### Strengths and limitations

This study has a number strengths and limitations. To our knowledge, this study is the first to systematically examine the use and quality of fidelity measurements in school-based anxiety, depression and suicide prevention programmes. Another strength of this study is that the screening of the literature was conducted by two researchers, with a third researcher consulted to reach a consensus when deemed necessary. In the process of data extraction, the models proposed by Carroll et al. [12] and Dusenbury et al. [9] were used to conceptualise the fidelity components. Furthermore, it should be noted that not all studies include the use of fidelity measurements in their title or abstract, which may result in studies being overlooked. Nevertheless, the collection obtained after screening is of such significance that we believe this review provides a comprehensive overview of fidelity measurements in school-based anxiety, depression and suicide prevention programmes. Also, even though a second reviewer assessed a percentage of studies to ensure consistency, no calculation of a formal inter-rater reliability made. This could be considered a limitation in terms of methodological transparency.

### Implications

This review shows that the recognition of fidelity measurements in school-based anxiety, depression and suicide prevention programmes is not a novel concept. However, in order for the field to fully benefit from fidelity measurements, certain improvements must be made. Firstly, the varying feasibility of measuring different fidelity components underscores the necessity for the development and standardisation of practical, user-friendly tools. The use of the developed tools could go beyond the measurement of fidelity. They could help intervention developers in systematically designing a fidelity measurement protocol, by giving support on which components to prioritise, how to operationalise them, and how to establish good methodological quality and relevance within different implementation contexts. To enhance flexibility and consistency in these efforts, it is important to explore opportunities to standardise the approach to measurements such as using shared definitions or reporting guidelines that can be applied across diverse programme settings. Secondly, the lack of consensus on the definitions and operationalisation of fidelity components highlights the importance of establishing a unified framework that provides clear guidelines for their measurement. Such a framework would not only improve the comparability of findings across studies but also reduce the complexity researchers face when designing fidelity assessments. Thirdly, the integration of fidelity measurements as a central element of intervention research must be prioritised. By embedding fidelity as a core component in study designs, researchers can better ensure that outcomes are interpreted in light of how well interventions were implemented, leading to more accurate evaluations of programme effectiveness. Finally, even though there is recognition of fidelity measurements, it remains underemphasized in practice. Increasing greater awareness among researchers and stakeholders about the critical role fidelity plays in understanding and optimising programme outcomes is therefore essential. Together, these steps can contribute to the systematic, high-quality assessment of fidelity, enabling the field to enhance the effectiveness and scalability of interventions.

## Conclusion

This review shows that the measurement of fidelity in school-based anxiety, depression and suicide prevention programmes remains underutilised and is often of moderate or low quality. It is also demonstrated that there is limited consensus on the definitions of fidelity and its components. Besides that, there is variability in practical tools for assessing fidelity components, which could be the cause of the inconsistent quality of the measurements. This also makes the comparison of fidelity measurements challenging. These findings emphasise the need for embedding fidelity as an integral part of intervention research. This can ensure a better understanding of why and how interventions are effective in differing contexts. Instead of developing a standardised tool, efforts could be made to standardise the approach to fidelity measurements with shared definitions and reporting guidelines, applicable across diverse interventions. Acknowledgement of the role of fidelity in optimising programme effectiveness is crucial for the field to move towards more consistent and high-quality implementation research.

## Supplementary Information

Supplementary Material 1.

Supplementary Material 2.

Supplementary Material 3.

## Data Availability

Data is provided within the manuscript or supplementary information files.

## Abbreviations

STBs: Suicidal thoughts and behaviours
PRISMA: Preferred reporting items for systematic reviews and meta-analysis NA: not applicable
RCTs: Randomised controlled trials
