# Supplementary material for: Assessing fidelity measurements in school-based anxiety, depression and suicide prevention programs: a systematic review
Source: BMC Public Health. 2025 Sep 1;25:3002. doi: 10.1186/s12889-025-24219-5 (PMC12400755; doi:10.1186/s12889-025-24219-5)
Supplement: Supplementary file 1 — Supplementary Material 1. [file 12889_2025_24219_MOESM1_ESM.docx]

**Table S2.** Table of full search strategy (on November 13^th^ 2023)

| **MEDLINE** |  |  |
| --- | --- | --- |
| **SUBJ. HEAD.** |  |  |
| 1 | "School Based Intervention"/ or "School Based Mental Health Services"/ or "School Counseling"/ or "Early Intervention"/ or “Student Health Services”/ | 3260 |
| 2 | "Major Depression"/ or exp "Self Destructive Behavior"/ or "Suicidal Ideation"/ or "Suicide"/ or "Youth Suicide"/ or "Suicide Prevention"/ or Suicidality/ or “Primary Prevention”/ | 104487 |
| 3 | 1 and 2 | 97 |
| 4 | 1 or 2 | 107650 |
| **TI,AB*,*ID** |  |  |
| 5 | (school* or educ* or “school-based" or college).ti,ab,id | 1126835 |
| 6 | (adolescen* or teen* or youth* or child* or "young people").ti,ab,id | 1920371 |
| 7 | (suicid* or depress* or MDD or dysphor* or melanchol* or dysthymi* or "self-destruct*" or "self-harm" or "self-injur*" or "self-inflicted" or "self-mutilation" or "sadness" or "anxiety" or anxious* or lonel* or "internalizing").ti,ab,id | 806310 |
| 8 | (interven* or prevent* or counsel* or program* or psychotherap* or therap* or "early interven*" or "mental health service" or "prevention program*").ti,ab,id | 6642642 |
| 9 | 4 and 5 and 6 and 7 and 8 | **1884** |
| **PsycINFO** |  |  |
| **SUBJ. HEAD.** |  |  |
| 1 | "School Based Intervention"/ or "School Based Mental Health Services"/ or "School Counseling"/ or "Early Intervention"/ or “Student Health Services”/ | 41791 |
| 2 | "Major Depression"/ or exp "Self Destructive Behavior"/ or "Suicidal Ideation"/ or "Suicide"/ or "Youth Suicide"/ or "Suicide Prevention"/ or Suicidality/ or “Primary Prevention”/ | 202674 |
| 3 | 1 and 2 | 1200 |
| 4 | 1 or 2 | 243265 |
| **TI,AB,ID** |  |  |
| 5 | (school* or educ* or “school-based" or college).ti,ab,id | 1076259 |
| 6 | (adolescen* or teen* or youth* or child* or "young people").ti,ab,id | 1030835 |
| 7 | (suicid* or depress* or MDD or dysphor* or melanchol* or dysthymi* or "self-destruct*" or "self-harm" or "self-injur*" or "self-inflicted" or "self-mutilation" or "sadness" or "anxiety" or anxious* or lonel* or "internalizing").ti,ab,id | 591191 |
| 8 | (interven* or prevent* or counsel* or program* or psychotherap* or therap* or "early interven*" or "mental health service" or "prevention program*").ti,ab,id | 1443682 |
| 9 | 4 and 5 and 6 and 7 and 8 | **6498** |
| **Embase** |  |  |
| **SUBJ. HEAD.** |  |  |
| 1 | "School Based Intervention"/ or "School Based Mental Health Services"/ or "School Counseling"/ or "Early Intervention"/ or “Student Health Services”/ | 214886 |
| 2 | "Major Depression"/ or exp "Self Destructive Behavior[PvDD1] "/ or "Suicidal Ideation"/ or "Suicide"/ or "Youth Suicide"/ or "Suicide Prevention"/ or Suicidality/ or “Primary Prevention”/ | 224005 |
| 3 | 1 and 2 | 3325 |
| 4 | 1 or 2 | 435566 |
| **AB, TI** |  |  |
| 5 | (school* or educ* or “school-based" or college).ab,ti | 1536489 |
| 6 | (adolescen* or teen* or youth* or child* or "young people").ab,ti | 2437772 |
| 7 | (suicid* or depress* or MDD or dysphor* or melanchol* or dysthymi* or "self-destruct*" or "self-harm" or "self-injur*" or "self-inflicted" or "self-mutilation" or sadness" or "anxiety" or anxious* or lonel* or "internalizing").ab,ti | 1067522 |
| 8 | (interven* or prevent* or counsel* or program* or psychotherap* or therap* or "early interven*" or "mental health service" or "prevention program*").ab,ti | 9062543 |
| 9 | 4 and 5 and 6 and 7 and 8 | **3833** |
| **ERIC** |  |  |
| **SUBJ. HEAD.** |  |  |
| 1 | "School Based Intervention"/ or "School Based Mental Health Services"/ or "School Counseling"/ or "Early Intervention"/ or “Student Health Services”/ | 14278 |
| 2 | "Major Depression"/ or exp "Self Destructive Behavior"/ or "Suicidal Ideation"/ or "Suicide"/ or "Youth Suicide"/ or "Suicide Prevention"/ or Suicidality/ or “Primary Prevention”/ | 6823 |
| 3 | 1 and 2 | 160 |
| 4 | 1 or 2 | 20941 |
| **TI,AB,ID** |  |  |
| 5 | (school* or educ* or “school-based" or college).ti,ab,id | 1138705 |
| 6 | (adolescen* or teen* or youth* or child* or "young people").ti,ab,id | 405460 |
| 7 | (suicid* or depress* or MDD or dysphor* or melanchol* or dysthymi* or "self-destruct*" or "self-harm" or "self-injur*" or "self-inflicted" or "self-mutilation" or "sadness" or "anxiety" or anxious* or lonel* or "internalizing").ti,ab,id | 31302 |
| 8 | (interven* or prevent* or counsel* or program* or psychotherap* or therap* or "early interven*" or "mental health service" or "prevention program*").ti,ab,id | 587286 |
| 9 | 4 and 5 and 6 and 7 and 8 | **919** |

**Total:** 13131

**Without duplicates:** 9996

**Duplicates removed**: 3135
