# Supplementary material for: Assessing fidelity measurements in school-based anxiety, depression and suicide prevention programs: a systematic review
Source: BMC Public Health. 2025 Sep 1;25:3002. doi: 10.1186/s12889-025-24219-5 (PMC12400755; doi:10.1186/s12889-025-24219-5)
Supplement: Supplementary file 3 — Supplementary Material 3. [file 12889_2025_24219_MOESM3_ESM.docx]

Additional file 3

| **Component** | **Number of studies (n)** | **References** |
| --- | --- | --- |
| **Adherence** | 57 | [18-26, 30-32, 34-44, 49-62, 64-68, 70-78, 81-86] |
| **Dosage** | 19 | [17, 19, 22, 24, 25, 27, 28, 30, 32-34, 45, 46, 65, 66, 69, 71, 80, 86] |
| **Quality of delivery** | 9 | [27, 28, 31, 39, 49, 50, 68, 70, 71] |
| **Responsiveness** | 24 | [17, 19, 20, 25, 29, 33, 47-50, 54-56, 63, 65, 66, 69-71, 79, 80, 86-88] |
| **Programme differentiation** | 0 | - |

**Table S3**: Fidelity components measured

| **Number of components measured** | **Number of studies (n)** | **References** |
| --- | --- | --- |
| **One** | 45 | [18, 21, 23, 26, 29, 35-38, 40-48, 51, 53, 54, 57-64, 67, 72-79, 81-85, 87, 88] |
| **Two** | 18 | [17, 20, 22, 24, 27, 28, 30-34, 39, 54-56, 68, 69, 80] |
| **Three** | 8 | [19, 25, 49, 50, 65, 66, 70, 86] |
| **Four** | 1 | [71] |
| **Five** | 0 | - |

**Table S4**: Number of Fidelity Components Measured per Study

**Table S5**: Characteristics of methods used to measure fidelity component

| Characteristics of method | Adherence (n) | Dosage (n) | Quality of Delivery (n) | Responsiveness (n) |
| --- | --- | --- | --- | --- |
| *Data collection method* |  |  |  |  |
| Observations^a^ | 41 | 5 | 9 | 1 |
| Logbook/checklist^b^ | 29 | 11 | 5 | 1 |
| Interviews^c^ | 3 | 0 | 0 | 2 |
| Questionnaires^d^ | 3 | 3 | 0 | 22 |
| *Subject of evaluation* |  |  |  |  |
| Student | NA | 15 | NA | 23 |
| Teacher | 57 | 5 | 9 | 5 |

*^a^ Observations refer to methods such as live or via audio- or video-recordings.*

*^b^ Logbooks* *and checklists refer to documents that varied in structure ranging from open-ended records, tick-box formats, and structured item lists*

*^c^ Interviews were used to explore fidelity components in a descriptive or reflective way*

*^d^ Questionnaires refer to structured self-report instruments completed by participants or implementers, often using fixed-response options*

**Table S6:** Overview of quality assessment criteria for measurement of fidelity components

| **Criterion** | **Number of studies (n)** | **References** |
| --- | --- | --- |
| **Adherence** |  |  |
| 1. Model used for evaluation | 13 | [18, 20, 21, 23, 42-44, 49, 50, 64, 72, 73, 86] |
| 2. Level of evaluation | NA |  |
| 3. Operationalisation fidelity component | 43 | [20-25, 30, 31, 34-37, 40-43, 49, 51-61, 64-67, 70, 72, 73, 76, 77, 81-86] |
| 4. Data collection methods | 17 | [20, 21, 36, 40-43, 52-56, 60, 68, 73, 81, 83] |
| 5. Quantitative fidelity measures | 35 | [18, 20-25, 30, 34-37, 40, 41, 44, 49-54, 57, 58, 61, 64-66, 68, 71-73, 77, 83, 84, 86] |
| 6. Frequency of data collection | 55 | [18-26, 30-32, 34-44, 50-62, 64-68, 70-78, 81-85] |
| 7. Relation fidelity component and programme outcome assessed | 4 | [22, 30, 49, 66] |
| **Dosage** |  |  |
| 1. Model used for evaluation | 1 | [34] |
| 2. Level of evaluation | 4 | [27, 28, 32, 86] |
| 3. Operationalisation fidelity component | 19 | [17, 19, 22, 24, 25, 27, 28, 30, 32-34, 45, 46, 65, 66, 69, 71, 80, 86] |
| 4. Data collection methods | 6 | [27, 28, 33, 65, 66, 86] |
| 5. Quantitative fidelity measures | 14 | [17, 19, 22, 24, 25, 30, 34, 45, 46, 65, 66, 71, 80, 86] |
| 6. Frequency of data collection | 17 | [17, 19, 22, 24, 25, 27, 28, 30, 32, 34, 45, 46, 65, 66, 69, 71, 86] |
| 7. Relation fidelity component and programme outcome assessed | 5 | [17, 22, 30, 46, 69] |
| **Quality of delivery** |  |  |
| 1. Model used for evaluation | 2 | [49, 50] |
| 2. Level of evaluation | NA | - |
| 3. Operationalisation fidelity component | 6 | [27, 28, 49, 68, 70, 71] |
| 4. Data collection methods | 2 | [28, 68] |
| 5. Quantitative fidelity measures | 5 | [28, 49, 50, 68, 71] |
| 6. Frequency of data collection | 8 | [27, 28, 31, 39, 50, 68, 70, 71] |
| 7. Relation fidelity component and programme outcome assessed | 2 | [10, 31, 49] |
| **Responsiveness** |  |  |
| 1. Model used for evaluation | 5 | [13, 19, 29, 48, 65, 86] |
| 2. Level of evaluation | 5 | [50, 55, 56, 63, 88] |
| 3. Operationalisation fidelity component | 22 | [17, 19, 25, 29, 33, 47-50, 54-56, 63, 65, 66, 69-71, 79, 80, 86, 87] |
| 4. Data collection methods | 1 | [20] |
| 5. Quantitative fidelity measures | 20 | [17, 19, 25, 29, 33, 47-50, 54-56, 63, 65, 66, 70, 71, 79, 80, 86] |
| 6. Frequency of data collection | 8 | [19, 20, 47, 55, 56, 63, 69, 88] |
| 7. Relation fidelity component and programme outcome assessed | 1 | [29] |
